# Draft Genome Sequence of the Histamine-Producing Bacterium Enterobacter kobei Strain 42-12

**DOI:** 10.1128/MRA.00760-19

**Published:** 2019-08-15

**Authors:** Chihiro Ohshima, Fumina Sato, Hajime Takahashi, Takashi Kuda, Bon Kimura, Zhi Hua Tao

**Affiliations:** a Department of Food Science and Technology, Faculty of Marine Science, Tokyo University of Marine Science and Technology, Tokyo, Japan; b Department of Food Science and Engineering, School of Light Industry and Chemical Engineering, Guangdong University of Technology, Guangzhou, China; University of Southern California

## Abstract

Bacterially produced histamine in food can be a cause of food poisoning. The whole-genome sequence is described for one histamine-producing Enterobacter kobei 42-12 isolate from the edible portion of salted, dried fish.

## ANNOUNCEMENT

Enterobacter kobei is a Gram-negative facultatively anaerobic motile oxidase-negative bacterium belonging to the Enterobacter cloacae complex (1). Species in the E. cloacae complex, including *E. kobei*, are popular as clinical isolates. They can be a cause of nosocomial infectious disease (2–4). Furthermore, these species have been isolated from food products (5, 6). Surveillance of histamine contamination in fish products was conducted. *E. kobei*, which is capable of producing histamine, was isolated during this surveillance. In order to investigate the development of the histamine-generating mechanisms, the whole-genome sequence of the isolate was identified.

Salted, dried fish, which is a popular food in China, Hong Kong, and Taiwan, was collected in a local market in Guangzhou City, China. A total of 10 g of the edible portion of the fish, mainly muscle, was cut and mixed with 90 ml histidine broth (pH 5.0) (7). This mixture was incubated at 30°C for 24 h, and the broth obtained was streaked onto Niven agar (pH 5.0) (8). The plates were then incubated at 30°C until colonies formed. After incubation, purple colonies, which indicate histamine production ability (8), were isolated. Genomic DNA from one of the isolates was extracted using a NucleoSpin tissue kit (Macherey-Nagel GmbH & Co.). Amplification and sequence identification of the 16S rRNA gene, *gyrB*, and *rpoB* were performed with the appropriate primer set (9–11). After sequencing, the sequence homology of each loci with *E. kobei* strains was analyzed by using the Genetyx ATCC module (Genetyx Co. Ltd.). As a result, the homology rate of the 16S rRNA gene with strain DSM 13645 was 98%. Additionally, the homology rate of *gyrB* with strain WCHEK045523 was 99%, and the homology rate of *rpoB* with strain ATCC BAA260^T^ was 99%. Then, the isolate was identified as *E. kobei*.

The isolate was cultured in histidine broth to the logarithmic growth phase and harvested using centrifugation (11,700 × *g*, 3 min). Genomic DNA was extracted from the pellet via the phenol-chloroform method (12). Following DNA extraction, the sequencing library was prepared with an Ion Xpress Plus fragment library kit (Thermo Fisher Scientific, Inc.). Subsequently, the prepared library was loaded onto an Ion 318 Chip by using an Ion Chef system, and sequencing was performed in an Ion Torrent Personal Genome Machine (PGM) (all Thermo Fisher Scientific, Inc.) based on the manufacturer’s protocol.

After sequencing, low-quality reads were filtered out and barcode sequences were trimmed using Torrent Server software (Thermo Fisher Scientific, Inc.) with default settings. A total of 1,926,314 reads were generated. The average read length was 220 bp. The assembler SPAdes v. 5.8.8.0 (13) was used with default parameters in the Torrent server to generate 31 contigs (≥500 bp) with a total length of 4,739,953 bp (the largest contig was 884,448 bp), a 54.86% GC content, and a 451,239-bp *N*_50_ value. The genome coverage was 49×. The assembled data were assessed using QUAST v. 2.3 (14) with default parameters. Genome annotation was conducted with the NCBI Prokaryotic Genome Annotation Pipeline (PGAP) at the National Center for Biotechnology Information (NCBI) (15). The present study will help uncover genes related to histamine production.

### Data availability.

The assembled genome sequence was registered in GenBank under the accession number BJEX00000000. The raw sequence reads have been deposited in the NCBI Sequence Read Archive (number DRR165933) and under the BioProject number PRJDB7859.
